# RNA Binding Sensitivity of Nonstructural Protein 8 Revealed by Small-Angle Neutron Scattering and Alphafold2 Prediction

**DOI:** 10.1021/acsnano.4c16790

**Published:** 2025-10-17

**Authors:** Xin Jiang, Jinxin Xu, Zhenyu Liao, Na Wang, Taisen Zuo, Changli Ma, Hanqiu Jiang, Yubin Ke, He Cheng, Howard Wang, Jinkui Zhao, Jun Fan, Jinsong Liu, Xiangqiang Chu

**Affiliations:** † Department of Physics, 53025 City University of Hong Kong, 83 Tat Chee Avenue, Kowloon, Hong Kong 999077, China; ‡ Shenzhen Research Institute, 53025 City University of Hong Kong, Shenzhen 518057, China; § Neutron Science Platform, Songshan Lake Materials Laboratory, Dongguan, Guangdong 523808, China; ∥ State Key Laboratory of Respiratory Disease, 74627 Guangzhou Institutes of Biomedicine and Health, Chinese Academy of Sciences, Guangzhou 510530, China; ⊥ China-New Zealand Joint Laboratory on Biomedicine and Health, Institute of Drug Discovery, Guangzhou Institutes of Biomedicine and Health, Chinese Academy of Sciences, Guangzhou 510530, China; ‡‡ Spallation Neutron Source Science Center, Dongguan, Guangdong 523803, China; ∇ China Guangdong Provincial Key Laboratory of Biocomputing, 74627 Guangzhou Institutes of Biomedicine and Health, Chinese Academy of Sciences, Guangzhou 510530, China; ○ China-New Zealand Joint Laboratory on Biomedicine and Health, 74627 Guangzhou Institutes of Biomedicine and Health, Chinese Academy of Sciences, Guangzhou 510530, China; ◆ Guangdong-Hong Kong-Macao Joint Laboratory of Respiratory Infectious Diseases, 74627 Guangzhou Institutes of Biomedicine and Health, Chinese Academy of Sciences, Guangzhou 510530, China; ¶ Beijing National Laboratory for Condensed Matter Physics, Institute of Physics, Chinese Academy of Sciences, Beijing 100190, China; †† Department of Materials Science and Engineering, 53025 City University of Hong Kong, 83 Tat Chee Avenue, Kowloon, Hong Kong 999077, China

**Keywords:** Small angle neutron scattering (SANS), Nonstructural protein 8 (Nsp8), RNA-protein complex, Thermal stability, Structural flexibility

## Abstract

The flexible structure enables nonstructural protein 8 (nsp8) to respond quickly to environmental changes, which are essential for RNA replication and transcription of SARS-CoV-2. In this work, small-angle neutron scattering and AlphaFold2 prediction were applied to characterize the structural change of SARS-CoV-2 nsp8 dimers and tetramers. The results demonstrated that the nsp8 tetramer with a more exposed core domain shows a low thermal stability. The exposed core domain increases its sensitivity to RNA and adapts its structure to interact with RNA. Our work reveals the structural difference between the two forms of SARS-CoV-2 nsp8s in the RNA synthesis process, which partly elucidates the molecular mechanism behind RNA replication of the RNA virus.

## Graphical abstract

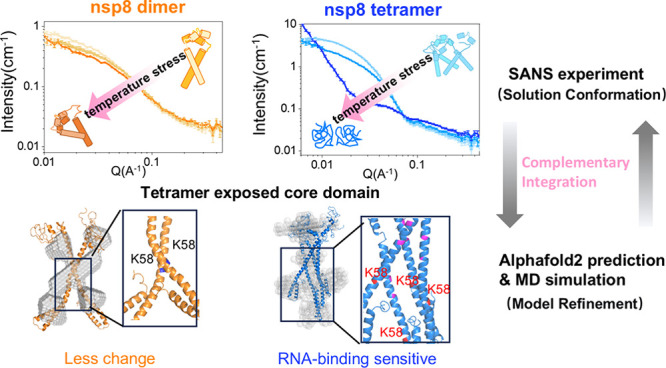

## Introduction

SARS-CoV-2 is a positive-strand RNA virus whose transcription and replication are mediated by an RNA-dependent RNA polymerase (RdRp) complex.^1^ The SARS-CoV-2 RdRp is a multisubunit replication-and-transcription complex formed by viral nonstructural proteins (nsp).^2^ Rapid incorporation of nucleoside/nucleotide analogs^3,4^ and association with non-nucleotide inhibitors of RNA polymerase complex both result in SARS-CoV-2 lethal mutagenesis,^5^ rendering RNA polymerase complex a promising therapeutic target for SARS-CoV-2.^6^ The Nsp family has two complexes with RNA polymerase activity. The first is nsp12-nsp7-nsp8, which provides the overall RNA-dependent RNA polymerase architecture.^7^ Its central unit is the nsp12 C-terminus, which contains the classical virus RdRp motif and mediates RNA elongation by a primer-dependent replication mechanism. In the nsp12-nsp7-nsp8 complexes of SARS-CoV^8^ and SARS-CoV-2,^9–11^ RNA elongation is mediated by nsp12, while nsp8 and nsp7 perform essential regulatory roles. The other nsp complex is the noncanonical putative primase nsp7-nsp8 complex^12^ unique to coronavirus, formed by nsp8 as the core molecule and nsp7.^13^ Compared with primer-dependent nsp12, nsp8 is capable of *de novo* RNA synthesis with low fidelity on ssRNA templates.^12^ Functional studies illustrated that the nsp7-nsp8 complex of SARS-CoV-2,^14^ SARS-CoV,^15,16^ and feline-CoV^17^ have putative primase activity for synthesizing new RNA chains. Nsp8 participates in the formation of both the nsp12-nsp7-nsp8 and nsp7-nsp8 complexes, which is crucial for RNA replication of SARS-CoV-2. However, conformational changes in nsp8 in the RNA synthesis process are still largely unknown.

As the name suggests, nsp8s are flexible nonstructural proteins, and many structural studies show that nsp8s have different conformations and perform various functions. Cryo-EM of nsp12-nsp7-nsp8 complexes of SARS-CoV^2^ and SARS-CoV-2 ^3^ shows a similar structure of the nsp7-nsp8 pair. In the complex, nsp8 cannot bring the amino acids essential for primase activity within adequate proximity of the nsp12 catalytic center,^2^ which proves nsp8 is a chaperone rather than a putative primase in the nsp12-nsp7-nsp8 complex. Nsp7-nsp8 primase complexes of SARS-CoV have two different constructs. One is a hollow, cylinder-like hexadecameric structure^15,18^ in which four nsp8 monomers adopt two distinct conformations, nsp8I and nsp8II. Nsp8I has a ‘golf club’- like structure composed of an N-terminal three-α-helix ‘shaft’ domain, a C-terminal three-α-helix domain, as well as a seven-strand β-sheet ‘head’ domain. Nsp8II has a golf club with a bent shaft.^15^ The other construct is a heterotetramer (2:2), in which the nsp8 scaffolds with putative head-to-tail interactions while the nsp7 subunits have no self-interaction.^19^ The primase of SARS-CoV-2 is remarkably different from SARS-CoV. Crystallographic and small-angle X-ray scattering (SAXS) results revealed that the nsp7-nsp8 complex of SARS-CoV-2 exists as a 2:2 heterotetramer whose core architecture is composed of two α-helix bundles.^13,14,20^ In addition, structural studies also proved that truncation of SARS-CoV nsp8 without the N-terminal long α-helix forms a tetrameric architecture similar to the structure of SARS-CoV-2 nsp7-nsp8 heterotetramer.^21^

In this work, we utilized small-angle neutron scattering (SANS), Alphafold2 prediction, dynamic light scattering (DLS), and circular dichroism (CD) to characterize the assembly pattern of nsp8 dimers and tetramers. Furthermore, we detected conformational change of nsp8 with rising temperature and RNA addition. Our data reveal that nsp8s form an unfolded but compact conformation to defend against temperature stress; the core domain, more exposed in a tetramer, is less stable than the dimer. When interacting with RNA, the Nsp8 tetramer with a more exposed core domain has a larger conformational change, which supports the idea that an Nsp8 tetramer is an RNA-sensitive form.

## Results and Discussions

### Nsp8 Dimers and Tetramers

From our earlier studies,^22^ nsp8 can assemble into two distinct types: the dimers and the tetramers. On this basis, we looked deeper into the structure and assembly pattern of the nsp8 dimer and tetramer. Size exclusion chromatography (SEC)-purified nsp8 dimers and tetramers were examined by DLS. The results revealed that their hydration kinetic radii (*R_H_*) were 28.29 ± 0.31 Å and 61.10 ± 0.72 Å, respectively. As the name ‘nonstructural protein’ (nsp) suggests, nsp8 exhibits significant structural flexibility, leading to a variety of conformations in solution. The DLS measurements reflect the *R_H_* of nonstructural protein as an ensemble average, encompassing the size distribution of these different conformations. To evaluate their temporal stability, we analyzed their particle size distributions after storing the nsp8 dimers and tetramers at 4 °C for one month; the *R_H_* of the dimers and tetramers is 26.61 ± 0.67 and 68.19 ± 0.95 Å (Figure 1A) (Table S1). No obvious changes are observed, indicating that both forms are stable with no mutual conversion in the given time range. CD spectra indicate a high degree of agreement between the secondary structures of the nsp8 dimers and tetramers, both of which are α-helical (Figure 1B).

**Figure 1:**
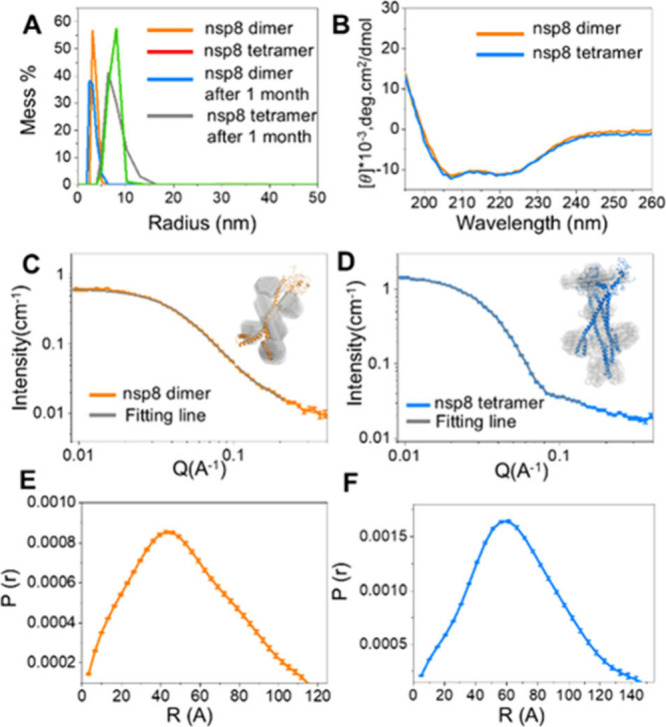
DLS spectra of the nsp8 dimer and tetramer after purification and storage for 1 month (A). CD spectra of nsp8 dimer and tetramer (B). SANS curve with coarse-grained models simulated by DAMMIN and the Alphafold2 prediction structure simulated by MD of nsp8 dimer (C) and tetramer (D) in 100% D_2_O buffer together with calculated SANS profiles from best-fitting atomic models (gray line). *P*(*r*) plot of nsp8 dimer (E) and tetramer (F).

The tertiary and quaternary structures of the nsp8 dimers and tetramers were studied by SANS experiments in D_2_O-based buffers. The concentration of the dimer sample is at 2.31 mg/mL, and the tetramer sample is at 1.92 mg/mL. The protein concentrations were determined by the BCA assay (Figure S1). Higher concentrations could cause liquid phase separation of nsp8, which is strongly reflected in the low *Q* region of the *S*(*Q*) scattering data. The radius of gyration (*R*_g_) obtained from Guinier plots of the scattering data using the ATSAS software^23^ is 36.92 ± 0.19 Å and 52.97 ± 0.17 Å for the nsp8 dimers and tetramers, respectively (Figure S6). The forward scattering intensity *I*(0) of the nsp8 tetramer is 2 times that of the dimer. Taking into account their differences in sample concentration, we obtain from Equation 1 ^24^ that the tetramer is twice as large as the dimer (Table 1), which agrees with their molecular composition and shows that both the tetramer and dimer samples are well-behaved monodisperse solutions.

1 $$I\left(0\right)=C_{p}\Delta \rho^{2}V_{p}^{2}$$

*C*_*p*_ is the concentration of the particle, Δρ is the scattering length density (SLD) contrast between buffer and particle, and *V*_*p*_ is the volume of the particle. The molecular weight is obtained from the scattering data, which are on the absolute scale^25^ (Figure 1C, D), using the ATSAS DATMW software package that utilizes a Bayesian consensus based on centration-independent molecular weight estimation.^23,26,27^ The molecular weights of the dimer and tetramer were estimated to be 41.93 ± 4.62 kDa and 90.67 ± 9.55 kDa by SANS data, which is consistent with the nsp8-His molecular weight of 22.98 kDa expected from the sequence (Table 1; Figure 1). The pair-distance distribution functions *P*(*r*) of the nsp8 dimer show characteristics of a rod-like particle (Figure 1E), suggesting that it adopts a more elongated shape. This is consistent with the results from protein crystallography studies.^21^ In contrast, *P*(*r*) for the nsp8 tetramers shows them to have a globular structural form (Figure 1F).

**Table 1:** nsp8 Dimer and Tetramer SANS Data Analysis

	Dimer	Tetramer
Concentration (mg/mL)	2.31	1.91
Points used for Guinier analysis	38	34
Guinier *R_g_* (Å)	36.92 ± 0.19	52.97 ± 0.17
*I*(0) (cm^–1^)	0.75 ± 0.21*10^–1^	1.61 ± 0.12*10^–1^
*D_max_* (Å)	101.26	158.33
MW estimation (kDa)	41.93 ± 4.62	90.67 ± 9.55

### Thermal Stability of the Nsp8 Tetramer Is Weaker than That of the Dimer

To assess the thermal stability of the nsp8 dimers and tetramers, we performed CD measurements at temperatures from 293 to 313 K to monitor their secondary structures. Figure S2 shows the thermal denaturation curves obtained from CD values with 220 nm light, which gives the estimated total α-helix content (Figure S2). The midpoint temperature (*T_m_*) value of the nsp8 dimer is 48.9 ± 0.7 °C, higher than that of the tetramer at 43.9 ± 0.9 °C. The overall shapes of the nsp8 dimers and tetramers were monitored using SANS measurements at temperatures of 288 K, 298 K, 308 K, and 318 K (Figures 2A and B). With changing temperatures, there is an obvious change in their *R_g_* , *I*(0) (Table S2), and radius of gyrations of cross-section *R_c_* (Figures 2C and D) (Table 2). *R_c_* can be calculated by equation 2.^28^

2 $$QI\left(Q\right)=I_{0}e^{-Q^{2}R_{c}^{2}/2}$$

**Table 2:** nsp8 Dimer and Tetramer Change with Temperature

	Dimer *R_g_* (Å)	Dimer *R_c_* (Å)	Dimer H (Å)	Dimer R (Å)
288 K	41.90 ± 0.67	9.89 ± 0.21	127.15 ± 7.45	16.79 ± 1.24
298 K	36.24 ± 0.51	8.07 ± 0.17	113.62 ± 6.31	15.36 ± 1.83
308 K	34.47 ± 0.47	7.25 ± 0.13	111.78 ± 5.25	11.52 ± 0.91
318 K	33.56 ± 0.36	6.93 ± 0.21	110.15 ± 4.43	10.21 ± 0.55

	Tetramer *R_g_* (Å)	Tetramer *R_c_* (Å)	Tetramer H (Å)	Tetramer R (Å)
288 K	55.08 ± 0.33	22.72 ± 0.45	141.38 ± 7.56	33.15 ± 1.31
298 K	53.27 ± 0.35	20.47 ± 0.60	137.45 ± 9.63	29.46 ± 1.03
308 K	50.71 ± 0.85	15.35 ± 0.19	137.38 ± 10.52	23.63 ± 0.49
318 K	--	4.24 ± 1.88	--	--

**Figure 2:**
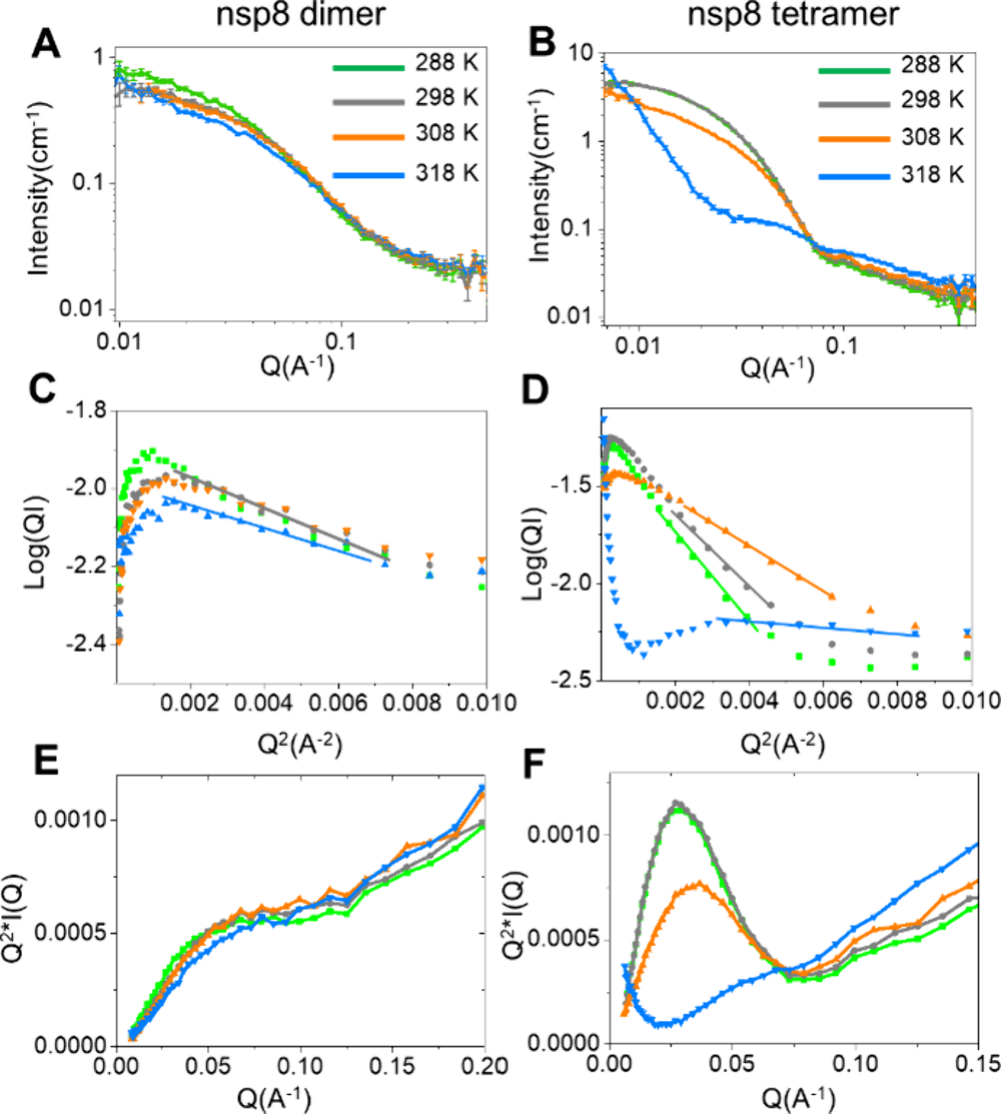
SANS spectra of nsp8 dimer (A) and tetramer (B); *R_c_* plot of dimer (C) and tetramer (D) with temperature. These analyses gave good straight-line fits over the *Q* range (0.03–0.08 A^–1^). The Kratky plots from SANS data of nsp8 dimer (E) and nsp8 tetramer (F).

Nsp8 dimers between 288 to 318 K and tetramers between 288 and 308 K exhibit a decrease in *R_g_* and *R_c_* values, which indicates that the nsp8s are tightly packed under the temperature stress, and an absorbing increase in *R_g_* of nsp8 tetramers in 318 K showed a tendency for aggregation, which also is presented by the DLS results (Figure S3). Furthermore, the SANS spectra of nsp8 tetramers cooling to 288 K from 318 K show the tetramers still aggregated, while nsp8 dimers almost recovered from the 318 K temperature stress (Figure S4). The CD spectra of nsp8 dimer and tetramer in 293 K cooling from 328 K also showed that the nsp8s aggregate, decreasing the overall CD signal significantly, and characteristic peaks of α-helix (208 nm) move to the characteristic peaks of unfolded random coils at around 200 nm (Figure S5). *I*(0) change can be attributed to a particle size and solute-excluded hydration layer.^29^ Nsp8 dimers between 288 to 318 K and tetramers between 288 to 308 K exhibit a decrease in *I*(0), indicating the solute-excluded hydration layer decrease, in agreement with increased protein hydration at low temperatures.^30^ The decrease in *I*(0) at 288–308 K could also result from a reduction in the particle volume (*V_p_*) of nsp8, as noted in Equation 1. The dramatic increase in tetramer *I*(0) at 318 K proves the particle size increase caused by aggregation. We also used DLS to monitor sample particle sizes. With varying temperatures, the obvious large-scale nsp8 tetramer aggregation was observed at 318 K, and we did not observe such dissociation, which would’ve resulted in smaller particles (Figure S3). We have performed cylinder model fitting and obtained the radius (*R*) and height (*H*) of the nsp8 dimers and tetramers at different temperatures (Figure S7, Table 2). In addition, the Kratky plot from SANS data was applied to check the globularity and flexibility of the nsp8 dimer and tetramer (Figures 2E and F). The Kratky plot analysis has already been corrected by subtracting the buffer as the background. The Kratky plot of the nsp8 tetramer exhibits a ‘bell-shaped’ peak at the low *q* region at 288 K, indicating a globular protein. At 318 K, the Kratky plot of the nsp8 dimer and tetramer has a plateau, indicating that both the nsp8 dimer and tetramer unfold with rising temperature. The MD simulation of nsp8 dimer and tetramer in different temperatures shows the dimer has relatively minor structural changes with temperature, with an RMSD of 19.49 Å in replica #1 and 18.10 Å in replica #2, while the tetramer undergoes more significant structural changes, with an RMSD of 33.83 Å in replica #1 and 44.84 Å in replica #2 (Figure S8).

The decrease in nsp8s in *R_g_* and *R_c_* suggested that both nsp8 dimer and tetramer would be more compact, and the plateau of the Kratky plot indicated unfolded states were induced by increasing temperature. And, nsp8 tetramers exhibit lower thermal stability and aggregate at 318 K.

### Nsp8 Tetramers Have an Exposed Active Core Domain

The structural changes of the nsp8 dimer and tetramer with RNA were detected by SANS spectra. We collected the SANS spectra of nsp8 dimers and tetramers with RNA at 1:1 and 1:2 molar ratios and got *R_g_* and *P*(*r*) functions (Figure 3A-D). The Nsp8 dimer and tetramer had a slight *R_g_* increase when in contact with RNA in a 1:1 molar ratio. With the addition of RNA in a 1:2 molar ratio, both the nsp8 dimer and tetramer showed more pronounced changes in SANS spectra. The *R_g_* and *P*(*r*) function shapes of the tetramers had a larger change than dimers, which indicated that nsp8 tetramers prefer to interact with RNA and induce more drastic conformational changes than dimers.

**Figure 3:**
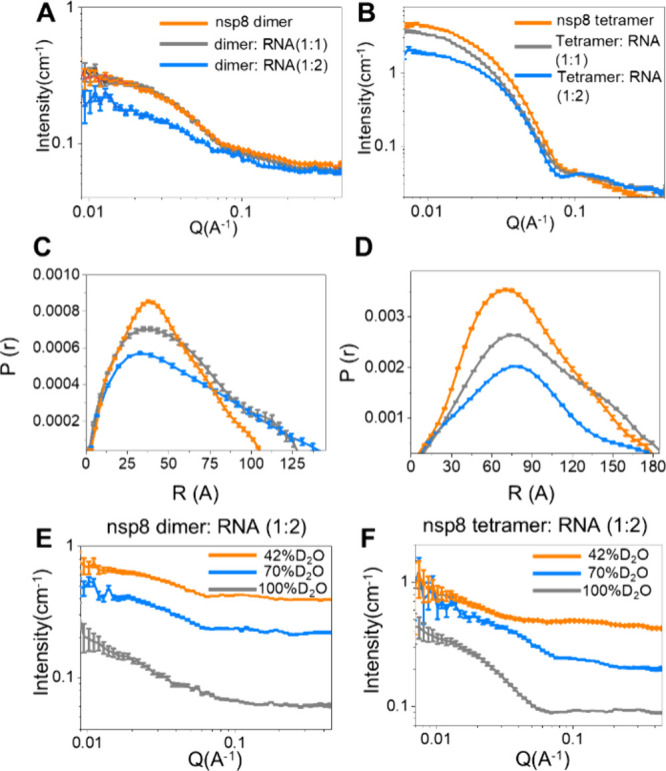
SANS spectra of nsp8 dimer (A) and tetramer (B) (orange) with the addition of RNA at 1:1 molar ratio (gray) and 1:2 molar ratio (blue). And the *P*(*r*) of nsp8 dimer (C) and tetramer (D). Nsp8 dimer (E) and tetramer (F) with RNA (molar ratio 1:2) in 100% D_2_O (gray), 70% D_2_O (blue), and 42% D_2_O (orange). The ssRNA sequence (GAGAAUGACAAA) employed in this work exhibits a relatively low molecular weight compared to nsp8s. It demonstrates low contrast in 42% D_2_O, resulting in weak scattering intensity; to facilitate visual comparison of their shapes, the scaling factor (10 for 70% D_2_O, and 100 for 42% D_2_O) was applied to make the *I*(0) in the same number scale. in Figures 3E and 3F, contrast matching was approached by mixing D_2_O/H_2_O solvent, which increases hydrogen content compared to pure D_2_O. The hydrogen enhanced incoherent scattering dominates at high *Q* where the coherent signal becomes negligible, which caused the flat high-Q background.

SLD contrast variation SANS adds information about the relative position and shape of the individual components within the complex. The inherent natural contrast between RNA and the protein can be utilized in the investigation of the RNA-binding state of nsp8 by SANS with a contrast match strategy. We acquired the SANS data of the nsp8 dimer and tetramer with or without the addition of RNA in 100% D_2_O, 70% D_2_O, and 42% D_2_O buffer (Figure 3E and 3F). A Stuhrmann plot is a quantitative way of describing the spatial distribution of contrast in a particle composed of several partners of different SLDs,^31^ which is a rapid and model-free method for determining whether RNA is located in the center or outside of the nsp8-RNA complex. The SLD contrast of nsp8–RNA complexes calculated from primary sequences to get the inverse of the total contrast (*Δρ*)^32^ and *R_g_* of the individual components of the nsp8-RNA complex derived from the *P*(*r*) functions were used for the calculation of the Stuhrmann plot as Equation 3.^31^ β relates to the separation of the mass centers of the two components. The nsp8-RNA complex is simple and homogeneously distributed; there is no separation between the centers of mass of nsp8 and RNA, and the above reduces to a linear equation when β equals 0. α relates to the distribution of scattering densities relative to the center of mass, which presents as the slope of the Stuhrmann plot, suggesting that, on average, the RNA, which has a higher SLD than the nsp8 protein, is in the internal region of the nsp8-RNA complex (Figure S9). The corresponding Guinier plots and *R_g_* values are shown (Figure S10 and Table S3. The above results supported a partition assembly with an ‘RNA inside, protein outside’ architecture, which is consistent with the latest model.^33^

3 $$R_{g}^{2}=R_{I}^{2}+\frac{\alpha }{\Delta \rho }-\beta /\Delta \rho^{2}$$

Δρ is the mean contrast for nsp8-RNA complex; *R*_*I*_ is *R*_*g*_ at infinite contrast; α relates to the distribution of scattering densities relative to the center of mass; β provides the separation of the mass centers of the two components.

SANS provides low-resolution structural information about the overall shape and size of nsp8 dimers and tetramers in solution but cannot precisely identify key amino acid residues and interfaces. To further explore the conformational difference between nsp8 dimers and tetramers, we used Alphafold2 to predict the possible structure of nsp8 dimers and tetramers. With the help of molecular dynamics (MD) simulation, we generated the models of nsp8 dimer and tetramer in solution buffer (Figure 4). We compared the MD-simulated models with SANS curve-simulated coarse-grained models (Figures 4E and F). The models showed that the nsp8 dimer has a cross-like structure, and the nsp8 tetramer has a loose structure. Compared to the dimer, the tetramer showed more obvious fitting deviations in the middle and high *Q* regions, which resulted from the higher structural flexibility of the tetramer. The tetramer exhibits significant conformational variability under different temperatures, suggesting higher structural flexibility. This flexibility arising from solvent interactions and disorder orientation can lead to deviations in MD-simulated models and SANS curves. Furthermore, SANS data represent an ensemble conformation, whereas the MD simulation reflects a limited conformational snapshot. Differences between the simulated structure and the ensemble-averaged experimental data are expected, particularly in flexible middle and high *Q* regions.

**Figure 4:**
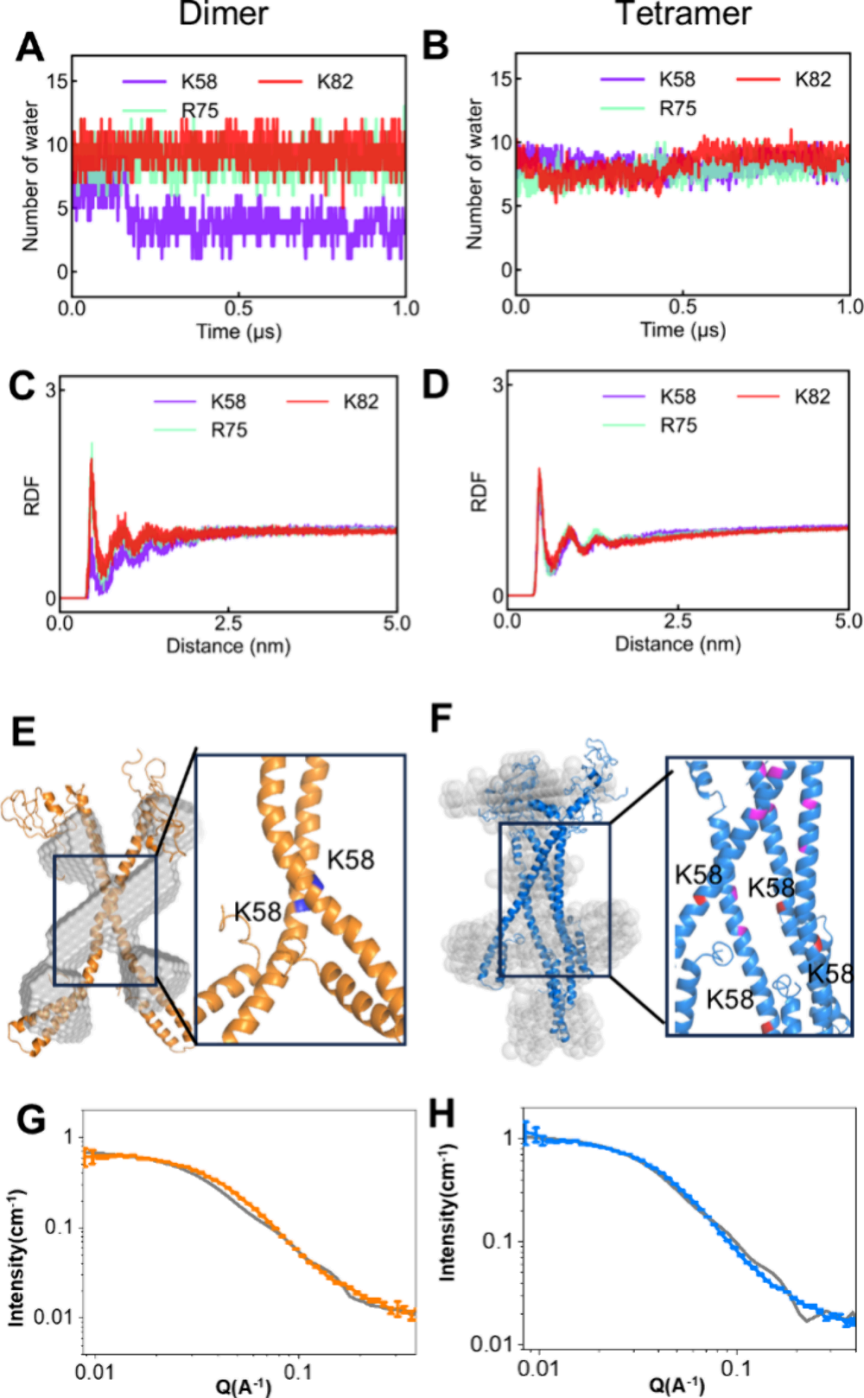
Number of water (A and B) and RDF (C and D) of the nsp8 residues K58, R75, and K82, which are critical for RdRp activity. The AlphaFold2 predicts models after MD simulation compared with the atomic models generated from SANS profiles of the nsp8 dimer (E) and tetramer (F). AlphaFold2-predicted models fit with experimental SANS data of nsp8 dimer (G) and tetramer (H) (χ^2^ = 17.61 and χ^2^ = 39.25 for dimer and tetramer, respectively).

It is reported that K58, R75, and K82 are critical for the RNA polymer activity of nsp8(13); we analyzed the RDF (radial distribution function) and the number of water molecules around these active residues. The results showed that K58, R75, and K82 of nsp8 tetramers and R75 and K82 of nsp8 dimers are almost the same, but the number of waters around K58 of nsp8 dimers is less than that of other residues, which revealed that the solvent exposure of active residues of nsp8 dimers is less than that of the tetramer. The above results indicated that the active residues of the tetramer have more solvent exposure than cross-like dimers, which makes the active core domain of the tetramer quickly interact with RNA.

In addition, the contrast matching SANS results demonstrated an altered shape of both the nsp8 dimer and tetramer with or without the addition of RNA, and the Stuhrmann plot suggested an ‘RNA inside, nsp8 outside’ structure, which corroborates well with the latest NMR results.^33^ The core domain exposed to the Nsp8 tetramer has a more obvious conformational change when interacting with RNA. The comparison of different forms of nsp8 gave insights into the dynamic structure changes of nsp8s with the environment. Our data propose a model that nsp8 tetramers, with a more exposed core domain, can facilitate their interaction with RNA and are sensitive to temperature change (Figure 5).

**Figure 5:**
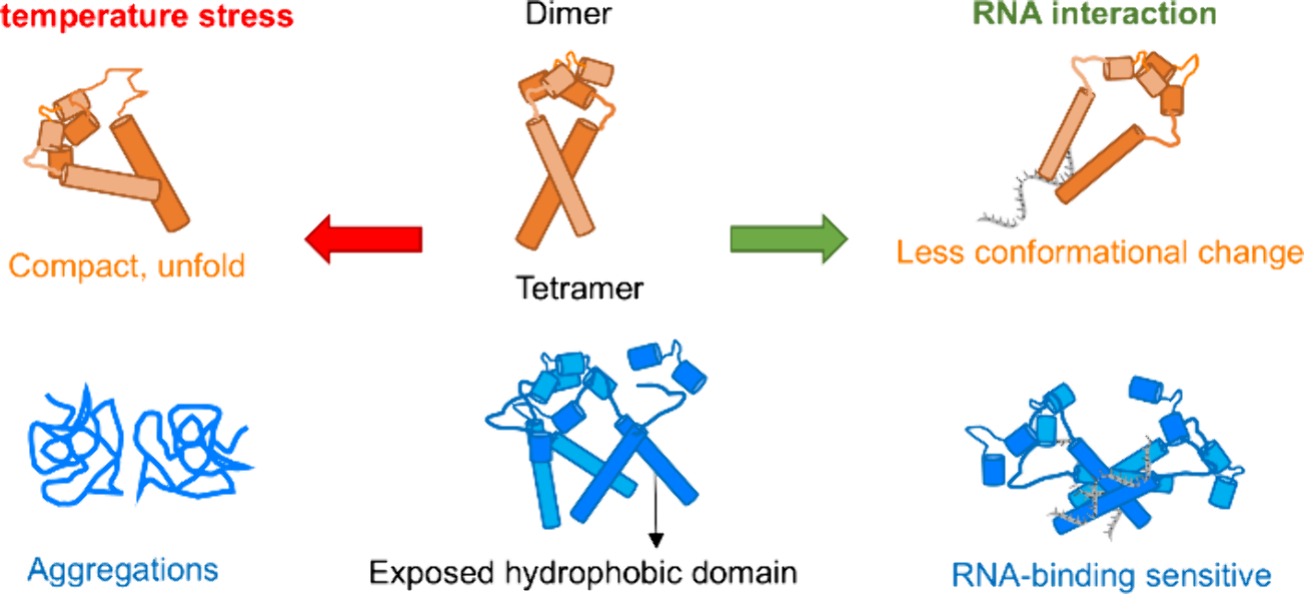
Nsp8 dimers (orange) have a linear structure. Nsp8 tetramers (blue) are globular and have more exposed core domains to interact with RNA (gray). With temperature rising, nsp8s form unfolded and compact states to pack the core domain and to resist temperature stress. Tetramers with more exposed core domains have less thermal stability and aggregate at high temperatures.

## Conclusions

In this study, SANS data of two different forms of nsp8, nsp8 dimer and tetramer, indicated that with the rising temperature, the size of nsp8 dimers and tetramers decreases and the structure unfolds, suggesting a compact unfolded state. RNA polymerase is sensitive to the temperature and is more active at high temperatures. However, higher reaction temperature increases its error rate.^34^ It is reported that temperature restricts the replication of SARS-CoV-2 in respiratory airway cultures,^35^ and the latest finding demonstrated that SARS-CoV-2 RdRp enzymatic activity is significantly enhanced from 33 to 37 °C.^36^ With the temperature stress, our SANS data showed the nsp8 tetramer would be more likely to unfold and aggregate, which indicated that the tetramer is more sensitive to temperature. In addition, Alphafold2 predicts that nsp8 dimers have a cross-like structure whose active residues were packed. The tetramers showed a more exposed structure, which has more water molecules around the active residues, facilitating their interaction with RNA.

In summary, we combined SANS and Alphafold2 prediction, revealing a model in which the flexible Nsp8 tetramer can respond to environmental changes and structurally adapt to changes to bind or release RNA, which provides conformational information in RNA synthesis of SARS-CoV-2 and other RNA viruses.

## Materials and Methods

### Sample Preparation

The SARS-CoV-2 nsp8 dimer and tetramer samples were prepared as previously reported.^22^ ssRNA used in this study was purchased from Genscript, China. The sequence is GAGAAUGACAAA.

### Small-Angle Neutron Scattering (SANS)

SANS measurements were performed at the China Spallation Neutron Source (CSNS). Nsp8 samples in 20 mM HEPES at pH 7.4 and 250 mM NaCl D_2_O buffer were measured in quartz cells with a 2 mm optical path length. The scattering experiment times of buffers and samples are 90 min. The empty beam and empty quartz cell were measured for 15 min for data reduction. Transmissions were measured for 10 min for each sample and the empty beam.

The presented SANS data were reduced and corrected for sample transmission, cell scattering, and detector background using reduction algorithms developed based on the Mantid framework^37^ provided by the instrument. The neutron scattering intensity was calibrated to absolute intensity using a secondary standard, Bates poly. The SasView (https://www.sasview.org/) software was employed to calculate the radius of gyration *R_g_* of all samples by the Guinier approximation.

SANS experiments with H/D-contrast variation were performed as above. The 70% D_2_O in the H/D contrast measurement was calculated using the neutron transmission data. Data analysis and size calculations were performed using the ATSAS^23^ software, including PRIMUS and GNOM.

### Dynamic Light Scattering (DLS)

Nsp8 dimer or tetramer with a concentration of 1 mg/mL was dissolved in a buffer containing 20 mM HEPES (pH 7.4) and 250 mM NaCl. The DLS analyses were performed by using DynaPro NanoStar (Wyatt Technologies). The quartz cells were filled with 100 μL of nsp8 sample solution for DLS measurement. The samples were measured through a 660 nm laser, θ = 90°. Ten successive measurements were performed per sample to get the average DLS data. The DYNAMICS software (Wyatt Technologies) was employed to analyze the DLS data to get the *R_H_* of all samples.

### Circular Dichroism (CD)

CD spectra of the nsp8 dimer and tetramer were monitored in a Chirascan V100 spectropolarimeter equipped with a Peltier temperature controller (PTC-423S) in a 2 mm path length cuvette. CD experiments were performed using a protein concentration of 20 μM dissolved in 20 mM HEPES at pH 7.4 and 250 mM NaCl in the range of 200 to 260 nm. To assess the thermal stability, the signal of ellipticities at 212 nm was monitored, and the temperature was increased linearly at a rate of 1 °C per min. Data from three independent experiments were used for the analysis.

### Structural Predictions Generated Using AlphaFold2

AlphaFold2 was extended to predict multiple-chain complexes, and the system was named AlphaFold-Multimer.^38^ We used extensive sampling approaches of AlphaFold2 ^39^ to blind predict full structures and yielded high-quality structural predictions of nsp8 dimers and tetramers.

### Molecular Dynamics (MD) Simulations

Coarse-grained structures and associated topologies of nsp8 dimers and tetramers were generated using the Martinize2 script.^40^ The Martini 3 force field^41^ was applied to the proteins, incorporating an elastic network with a spring constant of 700 kJ/mol and a cutoff distance of 0.9 nm for each monomer. Side-chain corrections were implemented. The proteins were positioned in a periodic boundary conditions (pbc) water box and solvated with Martini 3 water beads. Sodium and chloride ions were introduced to neutralize the system. Van der Waals (vdW) and electrostatic interactions were truncated at 1.1 nm with the reaction field method used for electrostatic interactions. Simulations were conducted under constant temperature/pressure (*NPT*) conditions using Gromacs 2020.^42^ The system temperature was controlled at 310 K using a velocity-rescale thermostat,^43^ and pressure was maintained at 1 bar with an isotropic Parrinello-Rahman barostat.^44^ All systems were minimized for 10,000 steps, with 1000 KJ·mol^–1^·nm^–2^ harmonic restraints applied to proteins. The systems were then heated to 310 K and equilibrated for 5 ns. Production simulations were performed for 1 μs without any restraint using a time step of 20 ps. Visualization and representation of the models were carried out using the Visual Molecular Dynamics (VMD) software.^45^ Analysis was conducted using built-in tools in Gromacs and locally written scripts.

## Supplementary Material



## Notes

Additional data and analysis (PDF)
