# Supplementary material for: RNA Binding Sensitivity of Nonstructural Protein 8 Revealed by Small-Angle Neutron Scattering and Alphafold2 Prediction
Source: ACS Nano. 2025 Oct 17;19(42):36876–83. doi: 10.1021/acsnano.4c16790 (PMC12574207; doi:10.1021/acsnano.4c16790)
Supplement: Supplementary file 1 [file nn4c16790_si_001.pdf]

RNA Binding Sensitivity of Non-structural Protein 8 Revealed by Small-Angle Neutron Scattering and Alphafold2 prediction

Xin Jiang [1,2], Jinxin Xu [3,4,6], Zhenyu Liao[9], Na Wang[3,6], Taisen Zuo[5], Changli Ma[5], Hanqiu Jiang[5], Yubin Ke[5], He Cheng[5], Howard Wang[2], Jinkui Zhao[2,8*], Jun Fan[9*], Jinsong Liu[3,4,6,7*], Xiangqiang Chu[1,10*]*

[1]Department of Physics, City University of Hong Kong, 83 Tat Chee Avenue, Kowloon, Hong Kong 999077, China

[2]Neutron Science Platform, Songshan Lake Materials Laboratory, Dongguan, Guangdong 523808, China

[3]State Key Laboratory of Respiratory Disease, Guangzhou Institutes of Biomedicine and Health, Chinese Academy of Sciences, Guangzhou 510530, China

[4]Graduate University of Chinese Academy of Sciences, Beijing 100049, China

[5]Spallation Neutron Source Science Center, Dongguan, Guangdong 523803,

[6]China Guangdong Provincial Key Laboratory of Biocomputing, Guangzhou Institutes of Biomedicine and Health, Chinese Academy of Sciences, Guangzhou 510530, China

China-New Zealand Joint Laboratory on Biomedicine and Health, Guangzhou Institutes of Biomedicine and Health, Chinese Academy of Sciences, Guangzhou 510530, China

[7]Guangdong-Hong Kong-Macao Joint Laboratory of Respiratory Infectious Diseases, Guangzhou Institutes of Biomedicine and Health, Chinese Academy of Sciences, Guangzhou 510530, China

[8]Beijing National Laboratory for Condensed Matter Physics, Institute of Physics, Chinese Academy of Sciences, Beijing 100190, China.

[9] Department of Materials Science and Engineering, City University of Hong Kong, 83 Tat Chee Avenue, Kowloon, Hong Kong 999077, China

[10]Shenzhen Research Institute, City University of Hong Kong, Shenzhen 518057, China

*Corresponding authors: xiangchu@cityu.edu.hk, liu_jinsong@gibh.ac.cn, jkzhao@iphy.ac.cn and junfan@cityu.edu.hk

SASBDB accession codes

The SANS data have been submitted to the Small Angle Scattering Biological Data Bank (SASBDB; http://www.sasbdb.org)[1] with accession codes SASDP66 (SARS-COV-2 nsp8 dimer), SASDP96 (SARS-COV-2 nsp8 tetramer).

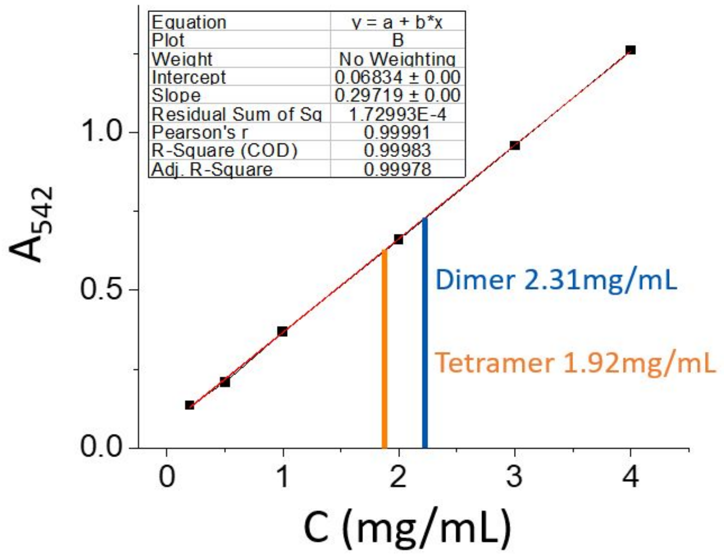

Figure S1: The concentrations of nsp8 dimer and tetramer were detected by BCA assay.

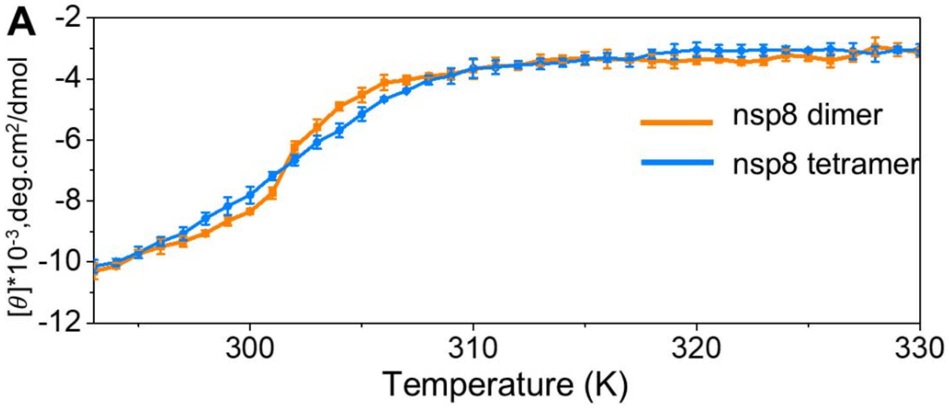

Figure S2: The thermal denaturation curves monitored by CD at 220 nm, reflecting the overall α-helical content. The midpoint denaturation temperature (T□) of the nsp8 dimer is 48.9 ± 0.7%˚C, which is higher than that of the tetramer (43.9 ± 0.9%˚C).

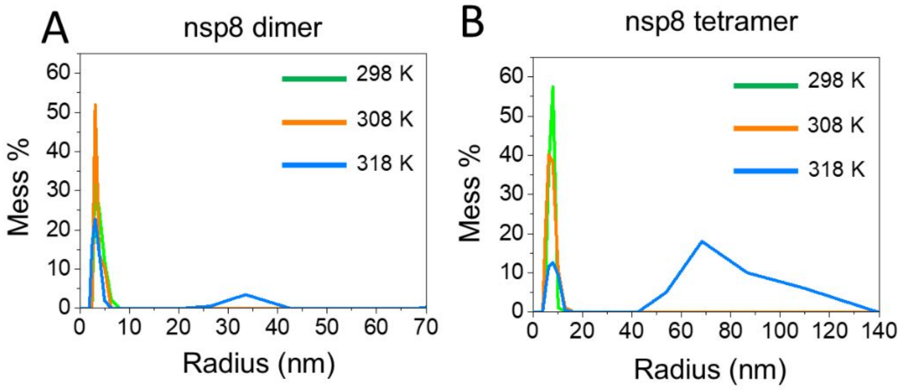

Figure S3: DLS spectra of nsp8 dimer(A) and tetramer (B) in 298K (green), 308K (orange), and 318K (blue). Nsp8 tetramers in 318 K showed a tendency for aggregation.

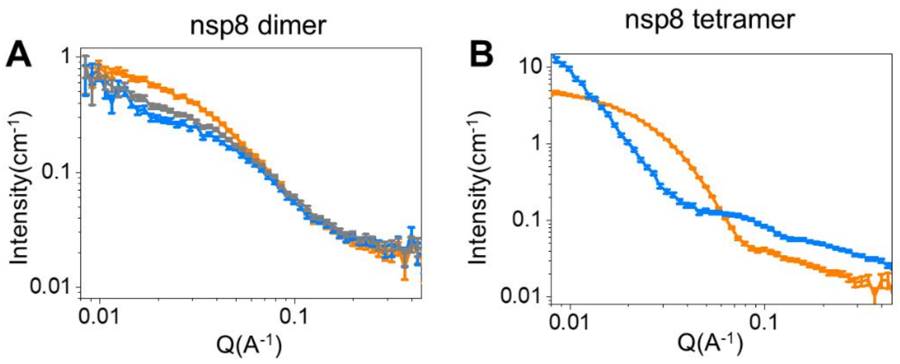

Figure S4: SANS spectra of nsp8 dimer in 288K (orange), 318K(blue), and back to 288K(gray)(A). and tetramer in 288K (orange) and 318K (blue) (B). SANS spectra show that nsp8 tetramers remain aggregated after cooling to 288%K from 318%K, while the dimers largely recover.

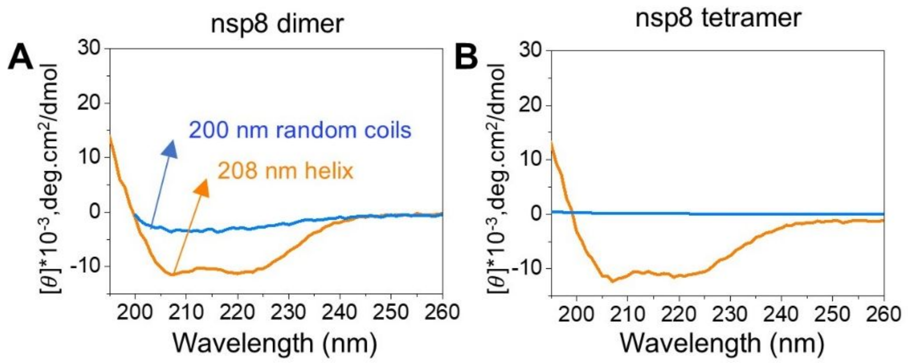

Figure S5: CD spectra of nsp8 dimer (A) and tetramer (B) in 293K (orange) and in 328K(blue). With the temperature stress, nsp8 dimerα-helix characteristic peaks (208 nm) move to the characteristic peaks of unfolded random coils at around 200 nm.

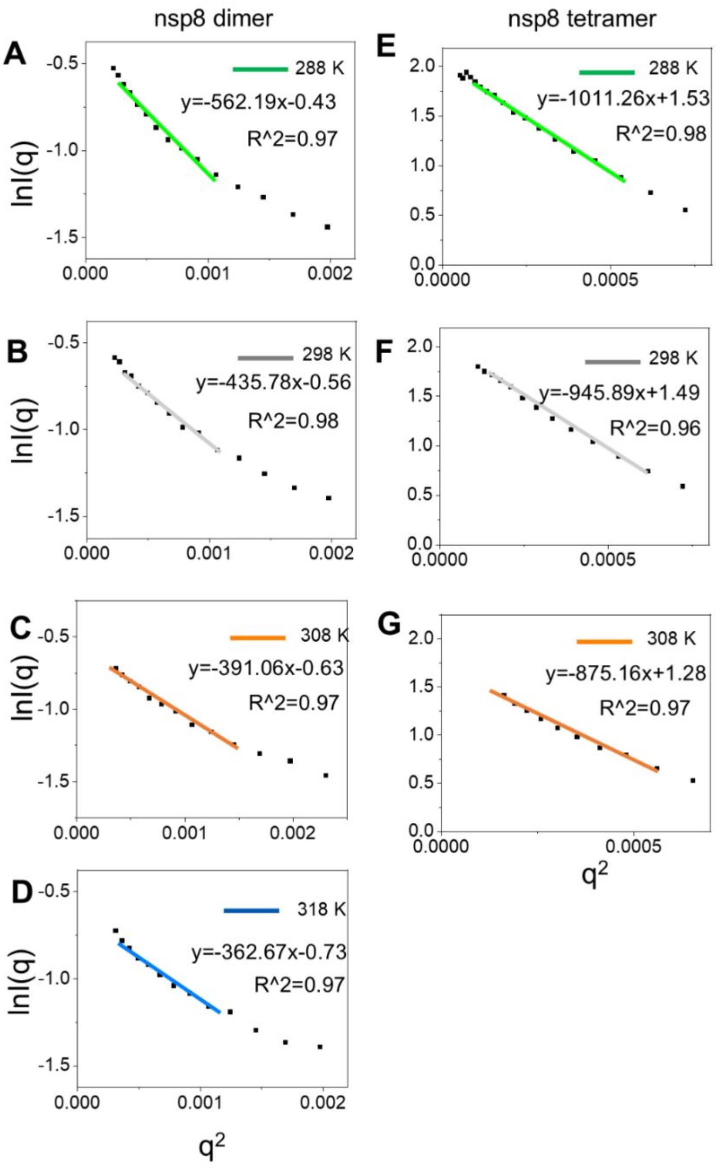

Figure S6: Guinier plots of SANS profiles of nsp8 dimer at 288 K ($q_{max}*R_g=0.031*41.06=1.29<1.3$) (A), 298 K ($q_{max}*R_g=0.035*36.12=1.27<1.3$) (B), 308 K ($q_{max}*R_g=0.037*34.24=1.28<1.3$) (C) and 318 K ($q_{max}*R_g=0.038*32.98=1.28<1.3$) (D) and tetramer at 288 K ($q_{max}*R_g=0.023*55.08=1.27<1.3$) (E), 298 K ($q_{max}*R_g=0.025*53.27=1.3$) (F), and 308 K ($q_{max}*R_g=0.025*50.71=1.27<1.3$) (G).

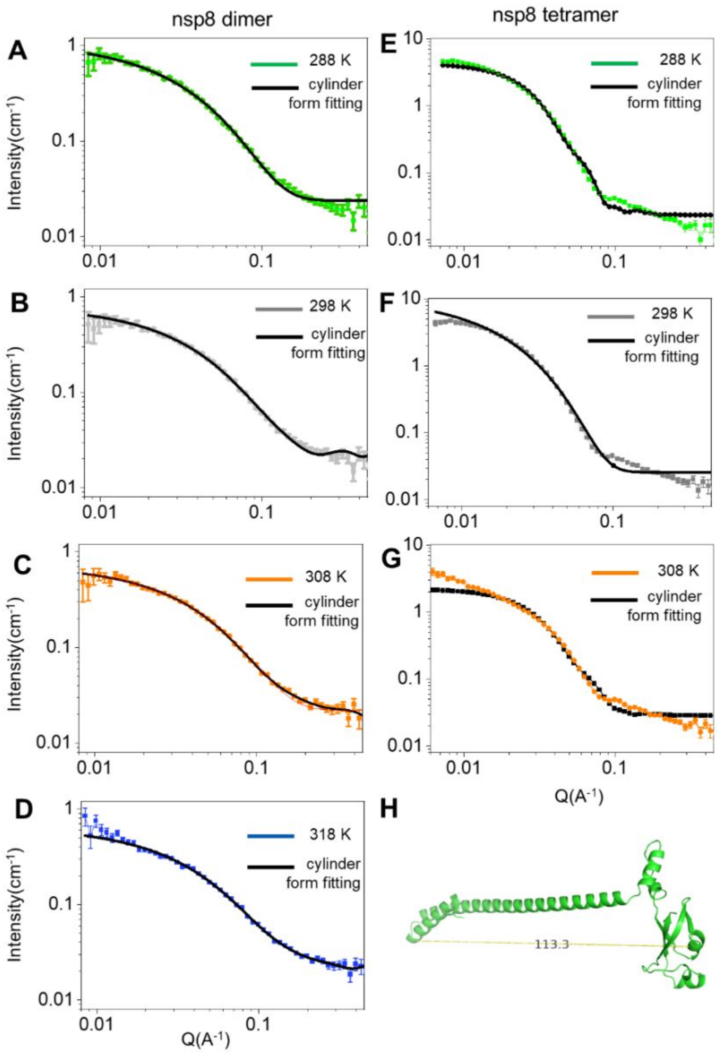

Figure S7: The SANS curve of nsp8 dimer in 288 K (χ^2=1.17) (A), 298 K (χ^2=0.97) (B), 308K(χ^2=1.09) (C), 318K (χ^2=1.21) (D) and nsp8 tetramer in 288 K (χ^2=1.86) (E), 298 K (χ^2=2.18) (F), 308K (χ^2=5.72) (G) with simple cylinder form fitting. And obtained the radius(R) and height (H) of the nsp8 dimers and tetramers at different temperatures (Table 2). The fitting results indicate that the height of nsp8 is approximately 110%Å, consistent with the predicted dimensions from AlphaFold-based ribbon representations (H).

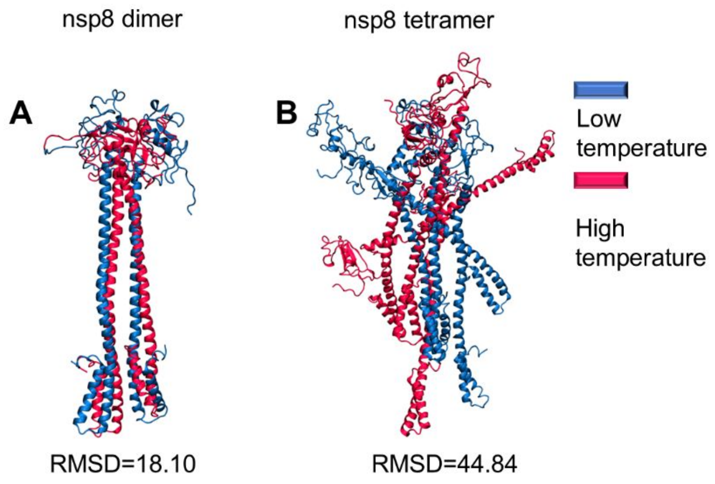

Figure S8: The comparison of nsp8 dimer (A) and tetramer (B) in high temperature (red) and low temperature (blue) MD simulations. The nsp8 dimer shows relatively minor structural changes with temperature, with an RMSD of 19.49 Å in replica #1 and 18.10 Å in replica #2, while the tetramer undergoes more significant structural changes, with an RMSD of 33.83 Å in replica #1 and 44.84 Å in replica #2.

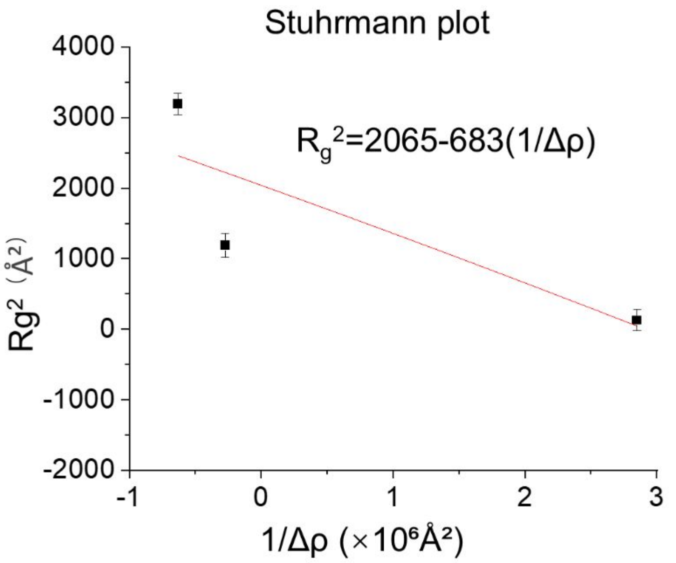

Figure S9: The Stuhrmann plot ($\Delta\rho$ in $\times 10^{-6}$ Å^{-2}, R_g in Å) of the nsp8 dimer obtained from the SANS dataset of the nsp8-RNA complexes in in 100% D_2O ($1/\Delta\rho$=-0.27×10^6 Å^2, R_g^2=1073.11±168.37 Å^2), 70% D_2O ($1/\Delta\rho$=-0.63×10^6 Å^2, R_g^2=3064.02±149.35. Å^2), 42% D_2O ($1/\Delta\rho$=2.8×10^6 Å^2, R_g^2=197.68±49.8Å^2). A straight line fitted to the data (R^2 = 0.78). The A positive slope in Stuhrmann plot indicates that the RNA, with its higher SLD, is predominantly located in the internal region of the nsp8–RNA complex.

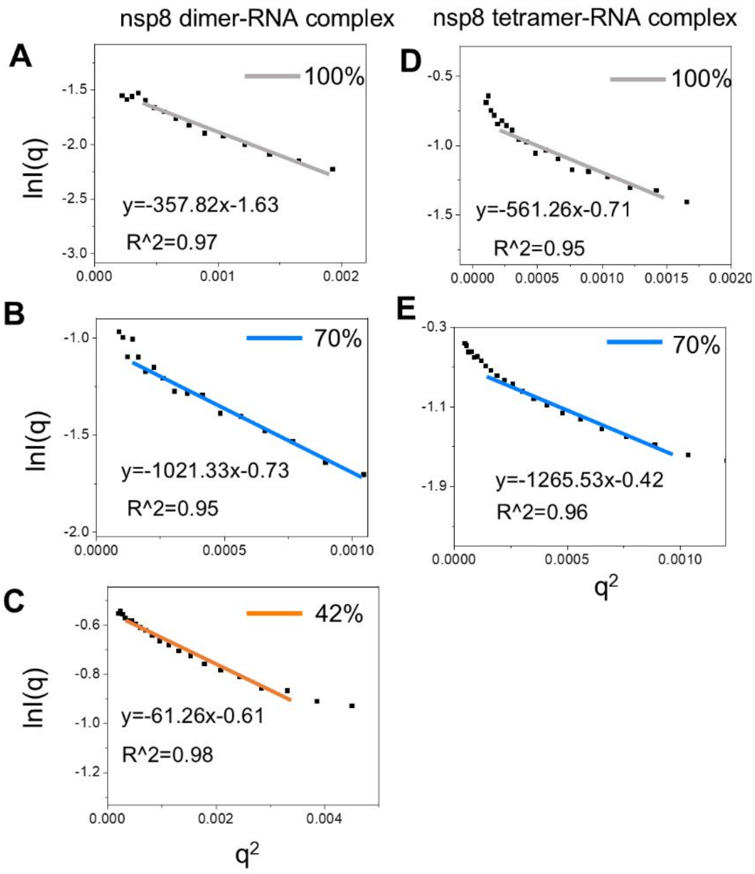

Figure S10: Guinier plots of SANS profiles of nsp8 dimer-RNA in 100% D_2O ($q_{max}*R_g=0.03*32.75<1.3$) (A), 70% D_2O ($q_{max}*R_g=0.02*55.35<1.3$) (B), 42% D_2O ($q_{max}*R_g=0.068*14.06<1.3$) (C), and tetramer-RNA complex in 100% D_2O ($q_{max}*R_g=$ $q_{max}*R_g=0.03*41.02<1.3$) (D), 70% D_2O ($0.02*61.62<1.3$) (E).

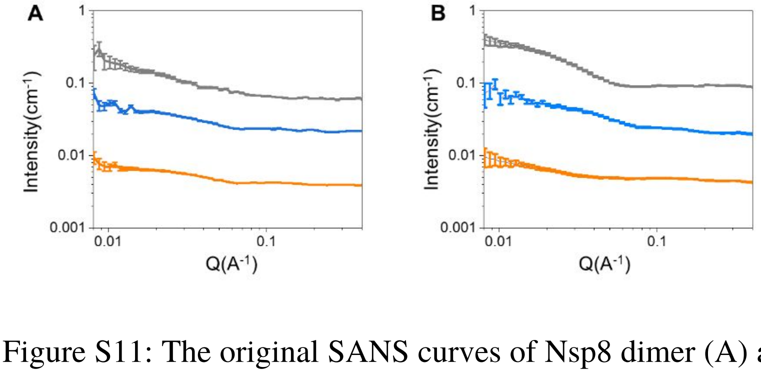

Figure S11: The original SANS curves of Nsp8 dimer (A) and tetramer (B) with RNA (molar ratio 1: 2) in 100% D_2O (grey), 70% D_2O (blue), and 42% D_2O (orange), with proper background subtraction.

Table S1: The DLS results of nsp8s before and after storage

	**R_H (Å)**	**R_H (Å) after one month storage**
**nsp8 dimer**	28.29±0.31	26.61±0.67
**nsp8 tetramer**	61.10±0.72	68.19±0.95

Table S2: The Rg and I(0) of nsp8 dimer and tetramer

	Dimer Rg (Å)	Dimer I(0)	Tetramer Rg (Å)	Tetramer I(0)
288K	41.06±0.57	0.72±0.04	55.08±0.33	4.61±0.23
298K	36.21±0.43	0.56±0.03	53.27±0.35	4.46±0.30
308K	34.24±0.41	0.53±0.03	50.71±0.85	3.63±0.49
318K	32.98±0.35	0.47±0.05	--	17.82±0.89

Table S3: The Rg of nsp8 dimer-RNA and tetramer-RNA complex in 100% D_2O, 70% D_2O, and 42%D_2O buffer

	SLD ($\times 10^{-6}$Å^{-2}) ($\times 10^{10}$ cm^{-2})	Dimer-RNA Rg (Å)	Tetramer-RNA Rg (Å)
100%D_2O	6.36	32.75±0.63	41.02±3.03
70%D_2O	4.28	55.35±1.25	61.62±2.35
42%D_2O	2.35	14.06±1.82	--

References

(1) Kikhney, A. G.; Borges, C. R.; Molodenskiy, D. S.; Jeffries, C. M.; Svergun, D. I., SASBDB: Towards an automatically curated and validated repository for biological scattering data. *Protein science : a publication of the Protein Society* **2020,** *29* (1), 66-75.
